# Metabolic Modulation in Dilated Cardiomyopathy: From Pathophysiology to Therapy

**DOI:** 10.31083/RCM45518

**Published:** 2025-11-27

**Authors:** Xiang Nie, Zhibing Lu

**Affiliations:** ^1^Department of Cardiology, Zhongnan Hospital of Wuhan University, 430071 Wuhan, Hubei, China; ^2^Hubei Provincial Clinical Research Center for Cardiovascular Intervention, 430071 Wuhan, Hubei, China; ^3^Institute of Myocardial Injury and Repair, Wuhan University, 430071 Wuhan, Hubei, China

**Keywords:** dilated cardiomyopathy, myocardial blood flow, energy supply and metabolism, genetic mutation

## Abstract

This review aims to synthesize current evidence on the role of cardiac energy metabolism in the pathogenesis of dilated cardiomyopathy (DCM), with a focus on myocardial blood flow, substrate utilization, genetic and metabolic pathways, and potential energy-targeted therapeutic strategies. DCM involves structural and functional impairments of the myocardium, often linked to genetic mutations (e.g., in titin (*TTN*) and *lamin*) or acquired factors, including infection, alcohol, drugs, and endocrine disorders. Moreover, the disruption of cardiac energy homeostasis is central to the pathogenesis of DCM, characterized by compromised energy supply, altered substrate metabolism, and reduced adenosine triphosphate (ATP) production, all of which collectively contribute to contractile dysfunction and disease progression. Emerging evidence indicates that impaired myocardial energetics, including reduced coronary blood flow, shifts in fuel utilization, and dysregulation of energy metabolic pathways, are hallmark features of DCM. Nonetheless, energy deficiency is increasingly being recognized as a key driver of DCM development and heart failure. Cardiac energy metabolic disruption is intimately involved in the pathophysiology of DCM and represents a promising target for novel therapeutic interventions. Current management strategies often overlook metabolic aspects; therefore, this review highlights the need to integrate energy-based approaches into the treatment paradigm for DCM.

## 1. Introduction

Dilated cardiomyopathy (DCM) is the third most common cause of heart failure and the most common cause for heart transplantation worldwide. DCM is characterized by left ventricular dilatation and contractile dysfunction in the absence of abnormal loading conditions and coronary artery disease [1, 2]. The etiologies of DCM are complex, involving both environmental and genetic factors, with over sixty genes encoding proteins for the cytoskeleton, sarcomere, and nuclear envelope known to be involved in the pathogenesis of DCM [3, 4]. Furthermore, genetic mutation may account for 20–48% of all cases of DCM. The genes involved in DCM appear to encode two major subgroups: the cytoskeleton or sarcomere. Currently, the identified cytoskeletal proteins include dystrophin, desmin, lamin, and metavinculin, among others; the sarcomere-encoding genes include β*-myosin heavy chain*, *myosin-binding protein C*, α*-tropomyosin*, *cardiac troponin T*, *cardiac troponin C*, and Z-disk proteins [5, 6, 7]. These genetic mutations primarily affect cardiac force generation and force transmission, eventually leading to cardiac contractile dysfunction.

Acquired causes of DCM could result from infection, alcohol abuse, drugs, and endocrine disturbances. Meanwhile, the infectious causes of DCM are various, but include viral, bacterial, fungal, and parasitic infections. Myocarditis, a frequent cause of DCM and heart failure, usually results from cardiotropic viral infection followed by active inflammatory destruction of the myocardium [8, 9]. Polymerase chain reaction (PCR) technology has enabled the detection of enteroviruses, adenoviruses, and parvovirus B19 in patients with DCM [10, 11]. Chronic alcohol abuse is one of the leading causes of DCM, especially in men aged 30–55 who have been heavy consumers of alcohol for at least 10 years [12, 13, 14]. Here, alcohol metabolites, such as acetaldehyde, impair cellular mitochondrial respiration, leading to cardiac contractile dysfunction [15, 16]. Long-term use of drugs, such as cocaine and antidepressants or antipsychotic drugs, is also known to cause DCM [17]. Cocaine causes left ventricular dysfunction by increasing catecholamine release, which leads to myocyte death by damaging the mitochondria [18, 19]. In addition, many cancer patients treated with anthracyclines, such as doxorubicin, epirubicin, and idarubicin, develop DCM and heart failure [20, 21]. The underlying mechanism of this toxic cardiomyopathy might be associated with the generation of reactive oxygen species (ROS) and the disruption of mitochondrial function [22].

Force generation and transmission defects, calcium homeostasis disorders, and metabolic abnormalities are other major causes of DCM. As we know, the heart is an energy-dependent organ that consumes large amounts of energy in the form of adenosine triphosphate (ATP), primarily produced through oxidative phosphorylation in the mitochondria [23]. Furthermore, abnormal mitochondrial activity leads to a decrease in energy production, which can result in pathological conditions. Free fatty acids and glucose are major energy substrates for cardiac contractile function. Patients with DCM exhibit alterations in myocardial metabolism characterized by decreased fatty acid metabolism and increased myocardial glucose metabolism [24, 25]. However, the underlying mechanisms of energy metabolism involved in the pathogenesis of DCM are rarely elucidated. Thus, this review discusses the energy supply and metabolism processes, along with the related genotypes, in the pathogenesis of DCM (Table 1). A comprehensive understanding of the energy metabolism involved can aid in identifying new molecular targets for treating DCM.

**Table 1.** **The known causes of metabolic DCM**.

Mechanism	Specific alterations	Consequences for the heart
Impaired myocardial perfusion	Reduced coronary blood flow, and microvascular dysfunction	Limited delivery of oxygen and metabolic substrates (fatty acids, glucose) to cardiomyocytes.
Altered substrate utilization	• Shift away from fatty acid oxidation	Inefficient ATP production per molecule of oxygen consumed (decreased oxygen efficiency).
	• Increased reliance on glucose and ketones	
	• Overall reduced metabolic flexibility	
Mitochondrial dysfunction	• Disrupted electron transport chain	Drastically reduced capacity for oxidative phosphorylation and ATP synthesis.
	• Increased ROS production	
	• Impaired mitochondrial dynamics	
Genetic and molecular regulation	Mutations in genes encoding metabolic enzymes (e.g., PPARα pathway) and structural proteins with metabolic roles (titin, lamin)	Directly disrupts the expression and activity of proteins critical for energy homeostasis.

DCM, dilated cardiomyopathy; ATP, adenosine triphosphate; ROS, reactive oxygen species; PPAR, peroxisome proliferator-activated receptor.

## 2. Energy Supply and Metabolism in the Normal Heart

The heart continuously supplies the organs that depend on a high level of ATP with oxygen, nutrients, and hormones. Indeed, a persistent production of ATP in the myocardium is particularly required to ensure that the myocardium continues to exert its proper function. Sufficient myocardial perfusion through blood vessels, such as epicardial conduit arteries, arterioles, capillaries, and veins, is necessary for myocytes to generate an adequate amount of ATP. Pre-arterioles and arterioles mainly control coronary blood flow (CBF), also referred to as the microvasculature [26]. Intramyocardial arterioles (<100 mm in diameter) have the highest resistance and respond either by myogenic control or metabolites. Under pathological conditions, coronary blood flow (CMD) can result from an abnormal structure of the coronary microvasculature, including intimal thickening, VSMC (vascular smooth muscle cell) proliferation, and low capillary density. Meanwhile, impairment of coronary blood flow can result from extracoronary causes (short diastolic perfusion time), compressive forces generated in the myocardium, or an abnormal coronary function (impaired vasodilatation due to endothelial dysfunction) [27]. In recent years, functional and structural alterations in coronary circulation have been extensively published in patients with heart diseases, such as heart failure with reduced ejection fraction (HFrEF), hypertrophic cardiomyopathy (HCM), and DCM [28, 29, 30].

During the neonatal period, the heart generates most of the ATP from glycolysis and lactate oxidation. However, as individuals age, the energy substrate of the myocardium shifts from glucose and lactate to free fatty acids (FFAs) [31]. These high-energy demands of the heart are met by the oxidation of fatty acids (FAs) and glucose in the mitochondria. FFAs are the preferred energy substrate in the healthy adult heart, supplying about 40%–90% of the total ATP, whereas glucose and lactate may provide additional energy [32, 33]. FAs generate more ATP per gram of substrate and require a greater rate of oxygen consumption for a given ATP synthesis rate than glucose. When the oxygen supply is insufficient, glucose can act as an ideal substrate for ATP production due to its lower oxygen consumption. Notably, almost two-thirds of the ATP hydrolyzed by the heart is used to fuel contractile work, with the remaining one-third used for ion pumps [34]. The myocardium in individuals with DCM prefers to generate ATP by using glucose as an energy substrate, resulting in an insufficient energy supply for cardiac contraction.

In general, normal coronary blood flow and sufficient energy substrates are necessary for maintaining cardiac function.

## 3. Myocardial Blood Flow Impairment in DCM: A Secondary Complication

In adulthood, energy substrates and nutrients essential for the survival and function of cardiomyocytes are predominantly supplied through the coronary blood vessels, including the coronary arteries and microvasculature. Stenosis of an epicardial coronary artery is usually considered the direct cause of angina and myocardial infarction, which are the leading causes of morbidity and mortality [35]. No coronary artery stenosis has been documented in DCM patients following coronary angiography [36]. However, impaired myocardial blood flow (MBF) is present in patients with DCM, which indicates that the microvasculature may be damaged. Previous studies have suggested that coronary microvascular dysfunction is associated with cardiovascular diseases such as heart failure with preserved ejection fraction (HFpEF) and HCM. Meanwhile, CMD may also contribute to the occurrence and progression of DCM [37, 38].

DCM is primarily defined by structural alterations such as left ventricular dilation and systolic dysfunction in the absence of significant coronary artery disease; however, impairments in MBF are increasingly recognized as playing a critical role in perpetuating and exacerbating the disease progression. Importantly, the blood flow abnormalities in DCM are distinct from those caused by epicardial coronary atherosclerosis. Instead, blood flow abnormalities primarily involve coronary microvascular dysfunction. This microcirculatory impairment is not the initial cause of DCM but rather a consequential pathology that arises from several mechanisms intrinsic to the dilated heart: (1) Microvascular compression: cardiac dilation and elevated ventricular wall stress directly compress the microvasculature, increasing resistance and reducing coronary flow reserve. (2) Endothelial dysfunction: the failing heart is characterized by neurohormonal activation, oxidative stress, and chronic inflammation, all of which impair the ability of the endothelium to regulate vasodilation. (3) Perivascular fibrosis: progressive interstitial fibrosis alters the mechanical environment around the microvessels, further hindering their capacity to dilate and augment flow. Therefore, while the etiology of DCM excludes obstructive coronary artery disease, the subsequent development of microvascular dysfunction promotes a vicious cycle of impaired blood flow, which limits the delivery of energy substrates, aggravates cardiomyocyte hibernation and injury, and ultimately contributes to the worsening of cardiac function. Therefore, assessing coronary microvascular function provides valuable prognostic information and may identify novel therapeutic targets for a patient population already established to possess DCM.

MBF impairment is present in patients with DCM as detected by echocardiography [39, 40, 41]. The resting MBF was shown to be comparable in DCM and healthy controls (1.13 ± 0.31 (DCM) vs. 1.14 ± 0.20 (control) mL/min/mL; *p* = not significant); meanwhile, hyperemic MBF under adenosine infusion was significantly impaired in DCM patients compared with controls (2.52 ± 1.29 (DCM) vs. 3.57 ± 0.88 (control) mL/min/mL; *p* = 0.014) [42]. Another study has also shown that resting the MBF is not significantly different in patients with DCM (0.48 ± 0.07 mL/min/g) and healthy controls (0.55 ± 0.19 mL/min/g; *p* = 0.41), whereas following dipyridamole administration, the increased MBF level observed in DCM patients was less pronounced than that in the controls (1.05 ± 0.35 (DCM) vs. 3.57 ± 0.88 (control) mL/min/mL; *p* = 0.014) [38]. These findings indicate that the resting MBF in DCM patients is not diminished compared with controls; however, stress levels after vasodilator administration were lower than those in the controls, which suggests that the MBF is impaired in patients with DCM [43, 44, 45].

Structural alterations of the coronary microvasculature are a direct cause of impairment to the MBF in patients with DCM. Vascular resistance is regulated through various mechanisms to match blood flow with oxygen demand. Meanwhile, low capillary density in the heart is a direct cause of MBF impairment. Histological sections from resected hearts of patients with DCM revealed that the average number of capillaries is approximately 2000 per mm^2^ in healthy controls and decreases to 1590 per mm^2^ in DCM patients [46]. Vascular endothelial growth factors (VEGFs) are prime regulators of angiogenesis, and knockout of VEGF-A may impair angiogenesis, leading to ischemic cardiomyopathy [47]. The mRNA transcript levels of *VEGF-A* and *VEGF-B*, as well as the protein levels of VEGF-A and VEGF-R1, were downregulated, consistent with the decrease in vascular density observed in DCM patients compared to controls [48]. However, VEGF-A knockdown did not elicit an angiogenic response in a unique mouse model of DCM caused by mitochondrial respiratory chain deficiency [49], suggesting that mitochondrial dysfunction may trigger a distinct pathway that leads to the progression of DCM. Coronary endothelial cells play key roles in angiogenesis. The coronary endothelium has multiple cellular origins during development, including the epicardium, sinus venosus, and endocardium [50]. During embryonic development, the heart development protein with epidermal growth factor-like domain 1 (HEG1) receptor is an important intercellular adhesion cadherin protein and maintains the function and integrity of blood vessels. Indeed, the loss of HEG1 in Zebrafish affected the stabilization of vascular endothelial cell connections and eventually led to pericardial edema and DCM [51]. In a rat model of DCM, the transplantation of pluripotent mesenchymal stem cells (MSCs) significantly increased capillary density in the myocardium, resulting in enhanced left ventricular function through the differentiation of MSCs into endothelial cells [52, 53].

In addition, the endothelium plays a crucial role in the tonic control of microvascular function by releasing vasodilator factors, including nitric oxide (NO) and prostaglandins. Functional polymorphisms in endothelial nitric oxide synthase (NOS3) are associated with a sevenfold increased risk of DCM compared to healthy controls [54]. Treatment with endothelium-dependent dilator acetylcholine and smooth muscle vasodilator adenosine was shown to increase the coronary flow reserve in normal patients but not in patients with DCM [55], suggesting impaired coronary microvascular function in DCM. Cardiac rehabilitation can enhance peripheral endothelial function and improve the cardiac function of DCM patients [56].

Microvascular dysfunction has also been identified in several inflammatory conditions, such as severe chronic periodontitis or inflammatory bowel disease [57]. Thus, inflammation may provide a mechanistic link between these comorbidities and cardiovascular events. Meanwhile, some interventional evidence suggests that anti-inflammatory biological therapies, such as anti-tumor necrosis factor treatments, can promote an improvement in coronary and peripheral microvascular dysfunction [58].

## 4. Energy Metabolism Substrates are Shifted in DCM

**Fig. 1.**
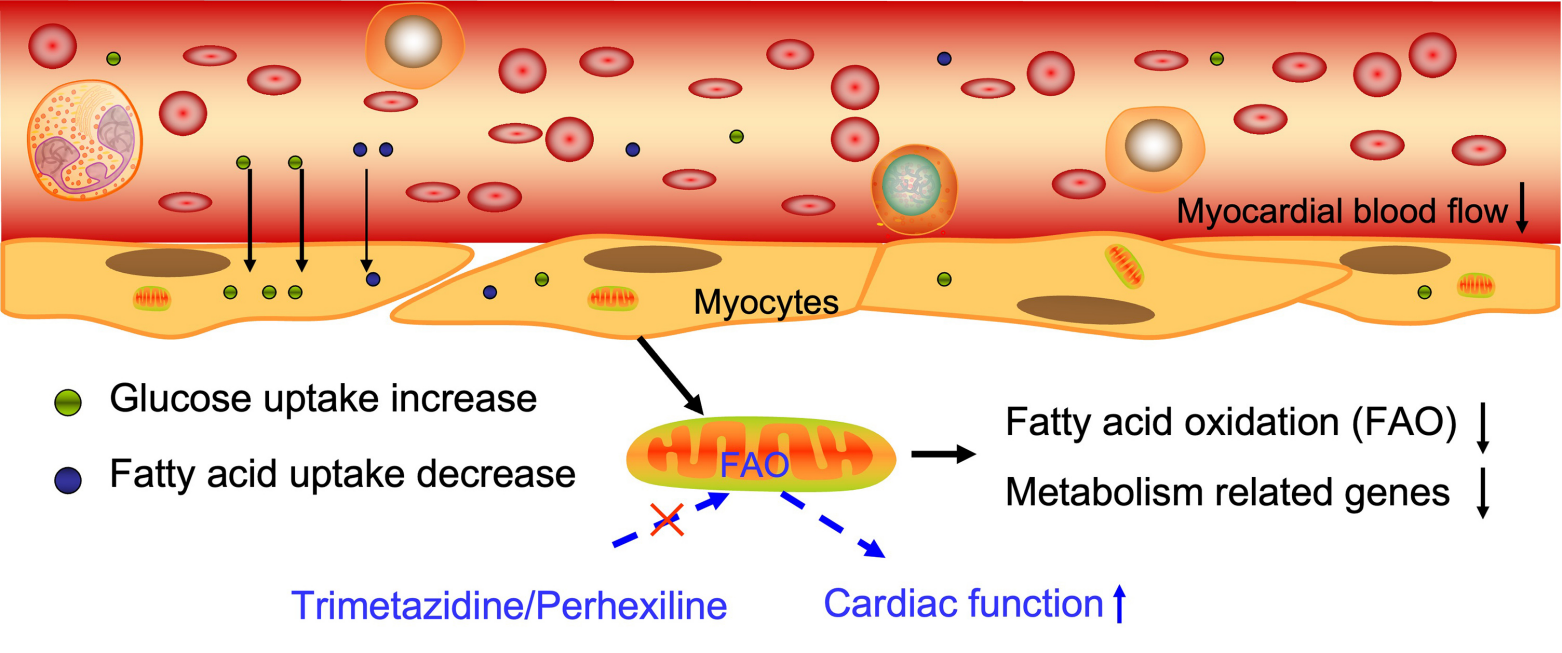
**The energy supply and metabolism process in DCM**. Myocardial blood flow is reduced in DCM patients, accompanied by an increase in glucose uptake and a decrease in fatty acid uptake. Fatty acid oxidation was reduced, and metabolism related genes expression were decreased in patients with DCM. The red cross markers means “inhibit”. The Figure was generated by Figdraw (HOME for Researchers, ZheJiang, China).

The heart prefers to utilize glucose as an energy source for ATP production in newborns to maintain cardiac structure and function, and shifts to FA metabolism in the adult myocardium, which provides approximately 60–90% of the required energy, with the remainder produced through glucose and lactate oxidation [59, 60]. Meanwhile, alterations in substrate utilization and oxidative stress are thought to account for contractile dysfunction and the progression of heart failure [23, 61]. The rate of myocardial FA utilization and oxidation is significantly lower in DCM patients than in the control groups, whereas the rate of myocardial glucose utilization is significantly higher [41, 62]. Myocardial FFA uptake is reduced and inversely associated with left ventricular ejection fraction in DCM patients [63]. Moreover, abnormal glucose tolerance in patients with DCM exacerbates the shift from FA to carbohydrate metabolism in the myocardium (Fig. 1).

In addition to mutations in structural proteins, mutations have been identified in genes associated with the metabolism of FAs and glucose. Indeed, long-chain 3-hydroxyacyl-CoA (LCHAD) is responsible for the beta oxidation of long-chain FAs in the mitochondrial membrane. A deficiency of LCHAD, which fails to metabolize long-chain FAs via beta oxidation, eventually leads to hypertrophic cardiomyopathy or DCM [64]. A neonate with a deficiency in glycogen branching enzyme may present symptoms of severe hypotonia and DCM in early infancy [65]. Mutations in the mitochondrial DNA (mtDNA) can cause several well-recognized human genetic syndromes characterized by deficient oxidative phosphorylation and may also play a role in DCM [66, 67, 68]. Mice deficient in the mitochondrial chaperone Hsp40 (heat-shock proteins) were also shown to develop DCM [69].

Thus, a metabolic shift from FAs to carbohydrates, combined with a failure to increase myocardial glucose uptake in response to loading conditions, may contribute to the pathophysiology of DCM. Therefore, treatments targeting substrate utilization and oxidative stress may be promising tools to improve cardiac function beyond that achieved with neuroendocrine inhibition.

## 5. Energy Metabolism-Related Genes and Pathways are Dysregulated in DCM

A significant proportion of DCM cases, particularly familial forms, are driven by pathogenic mutations in a diverse set of genes [70]. It is increasingly recognized that many of these culprit genes encode proteins that are intrinsically involved in cardiac energy metabolism, mitochondrial biogenesis, and oxidative phosphorylation. This genetic evidence provides a fundamental link, suggesting that metabolic impairment is not merely a secondary consequence of heart failure but can be a primary initiating event in the pathogenesis of DCM. Meanwhile, titin (TTN) is primarily a structural protein; however, mutations in the *TTN* gene represent the most common cause of familial DCM. Emerging evidence suggests that *TTN* mutations can disrupt the precise spatial organization of mitochondria within the cardiomyocyte, thereby impairing efficient energy production and distribution, which may contribute to disease progression [71]. Laminopathies result from mutations that severely disrupt nuclear integrity and gene expression, including the transcriptional programs governing peroxisome proliferator-activated receptor (PPAR) signaling and mitochondrial function. These result in a profound metabolic shift away from FA oxidation towards glycolytic metabolism, even in the adult heart, ultimately leading to an energy-deficient state [72, 73]. Mutations in the nuclear genes and mtDNA that encode mitochondrial proteins (e.g., those involved in the electron transport chain, mitochondrial dynamics, or coenzyme Q10 biosynthesis) can directly cause DCM [74].

**Fig. 2.**
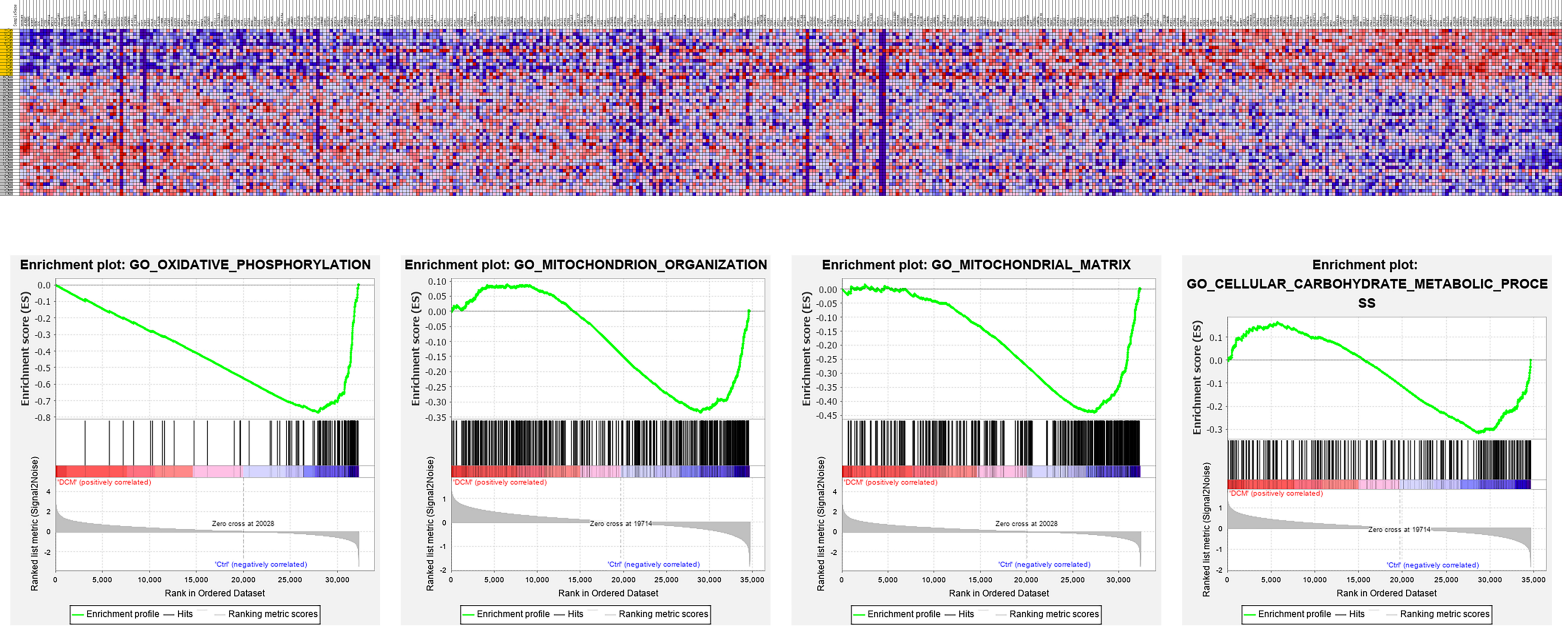
**The re-analysis of gene expression and pathways in heart tissue from DCM patients**. Pathways such as oxidative phosphorylation, mitochondrial organization, and mitochondrial matrix are dysregulated in DCM patients. The figure was generated using the GSEA 4.4.0 (UC San Diego, USA).

Additionally, mutations and dysregulation in genes encoding for the cytoskeleton and sarcomere are involved in the pathological process of DCM [75, 76, 77]. Differentially expressed genes have been identified between normal and DCM samples in several studies using RNA sequencing. Indeed, we previously collected and re-analyzed these gene expression profiles from the Gene Expression Omnibus database using gene set enrichment analysis (GSEA) (National Evaluation Series (NES) score >1.7 and *p* < 0.05) to explore the changes in energy metabolism related to DCM (GSE116250, GSE95140, GSE120852) [78, 79, 80]. Pathways such as pyruvate metabolism, glycogen metabolism, and mitochondrial calcium ion transport were significantly downregulated in DCM [80]. Impaired mitochondrial pathways involved in cardiac metabolism, including mitochondrial fusion, intrinsic components of the mitochondrial membrane, and mitochondrial gene expression, were also identified [81]. The dysregulation of mitochondrion-regulated genes and pathways (mitochondrial translational termination, mitochondrial protein complex, respiratory chain complex) has also been reported in DCM patients (Fig. 2) [82, 83]. These results indicate that energy metabolism-associated genes and pathways are dysregulated, which contributes to the pathophysiology of DCM.

## 6. Energy Metabolism-Based Treatment in DCM

Over the last few decades, treatment strategies for DCM have mainly focused on preventing cardiac remodeling and relieving the syndrome of heart failure; however, the three-year mortality rate remains high at 20% [84, 85].

**Fig. 3.**
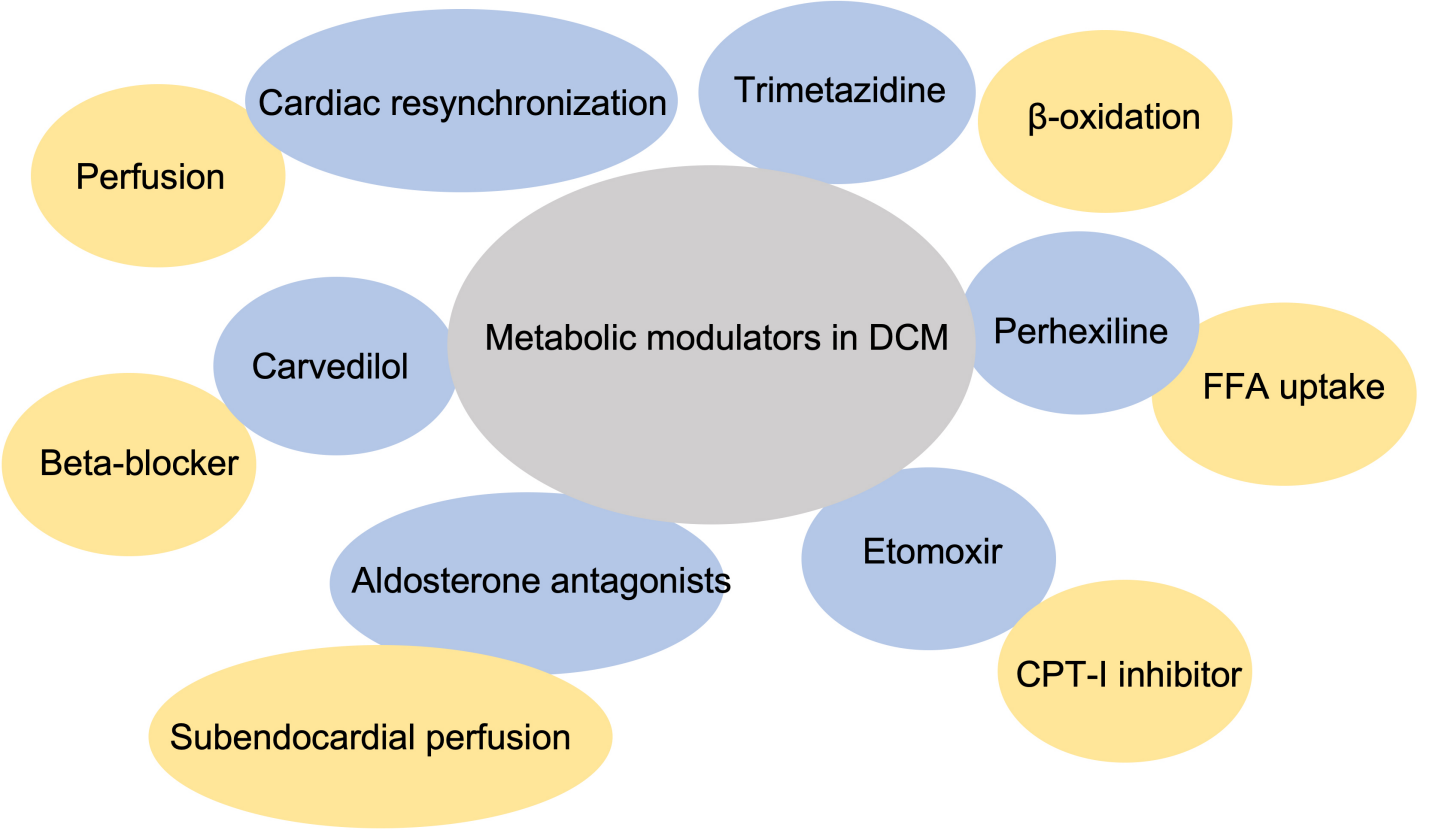
**Metabolic modulators in dilated cardiomyopathy**. FFA, free fatty acid; CPT-1, carnitine palmitoyl transferase-1.

Metabolic modulators have garnered significant attention as potential therapeutic agents for heart failure, particularly in DCM (Fig. 3). Among these, trimetazidine, an inhibitor of long-chain 3-ketoacyl coenzyme A thiolase, the final enzyme in mitochondrial fatty acid β-oxidation, has been shown to moderately reduce FFA oxidation and ameliorate insulin resistance without altering the myocardial oxidative rate, thereby contributing to improved cardiac function [86, 87]. Similarly, perhexiline acts by inhibiting the uptake of mitochondrial FFA enzymes: carnitine palmitoyl transferase (CPT)-1 and CPT-2 [86]. Clinical studies have demonstrated that perhexiline treatment leads to notable enhancements in peak exercise oxygen consumption, quality of life, and left ventricular ejection fraction in DCM patients [88]. Another CPT-1 inhibitor, etomoxir, has also exhibited beneficial effects, significantly increasing both cardiac output during exercise and left ventricular ejection fraction [89].

In addition to metabolic modulators, established heart failure treatments, such as aldosterone antagonists, can improve subendocardial perfusion and correct supply–demand energy imbalances in nonischemic DCM. Additionally, carvedilol, a beta-blocker with multifaceted properties, can reduce the risk of hospitalization and mortality in patients with heart failure [90]. Recent evidence further indicates that long-term carvedilol therapy significantly increases coronary flow reserve and reduces the incidence of stress-induced perfusion defects [91]. Furthermore, cardiac resynchronization therapy has beneficial effects on cardiac function by increasing coronary flow reserve in patients with DCM [92].

These studies indicate that energy-based treatment promotes benefits for cardiac function through improving coronary microcirculation and energy substrate utilization.

## 7. Conclusions

DCM is the primary cardiomyopathy and a common cause of heart failure worldwide, the pathophysiology of which is complex, including genetic mutation and environmental mediators. Recently, genes involved in DCM have been extensively studied, and acquired factors, such as viral infections and certain drugs, have also been investigated. However, energy supply and metabolism in the heart of DCM have yet to be systematically elucidated. Studies indicate that coronary artery microcirculation in DCM is disturbed, leading to an insufficient oxygen and energy supply, which is inadequate for the myocardium. Cardiac remodeling and ventricular stiffness may constrict or partially block microcirculation, leading to a reduction in MBF. Cardiomyocytes preferentially utilize glucose as a substrate for ATP production over FAs in DCM. Other energy substrates, such as lactic acid and pyruvic acid, may also play a crucial role in supplying ATP to cardiomyocytes.

An adequate ATP supply is essential for cardiomyocytes; however, the energy substrate transformation process may also be significant for cardiac function. Moreover, energy metabolism-related genes are also dysregulated, especially in mitochondrial pathways in DCM. Energy metabolism-based treatments have garnered increasing attention and are being employed in clinical trials. Meanwhile, improving microvasculature using carvedilol and aldosterone significantly increases cardiac output in DCM. Inhibition of fatty acid oxidation, as seen with trimetazidine and perhexiline, has been shown to have beneficial effects on cardiac function and clinical outcomes. Microvasculature, fatty acid, and glucose metabolism may become therapeutic targets for DCM in the future.
